# Visualization and exploration of linked data using virtual reality

**DOI:** 10.1093/database/baae008

**Published:** 2024-02-23

**Authors:** Alexander J Kellmann, Max Postema, Joris de Keijser, Pjotr Svetachov, Rebecca C Wilson, Esther J van Enckevort, Morris A Swertz

**Affiliations:** Department of Genetics, University of Groningen, Antonius Deusinglaan 1, Groningen, Groningen 9713 AV, The Netherlands; Department of Genetics, University Medical Center Groningen, Antonius Deusinglaan 1, Groningen, Groningen 9713 AV, The Netherlands; Department of Genetics, University Medical Center Groningen, Antonius Deusinglaan 1, Groningen, Groningen 9713 AV, The Netherlands; Department of Genetics, University Medical Center Groningen, Antonius Deusinglaan 1, Groningen, Groningen 9713 AV, The Netherlands; Center of information technology, University of Groningen, Nettelbosje 1, Groningen, Groningen 9747 AJ, The Netherlands; Public Health, Policy & Systems, University of Liverpool, Block B, 1st Floor, Waterhouse Building, 1-5 Dover Street, Liverpool L69 3GL, United Kingdom; Department of Genetics, University of Groningen, Antonius Deusinglaan 1, Groningen, Groningen 9713 AV, The Netherlands; Department of Genetics, University Medical Center Groningen, Antonius Deusinglaan 1, Groningen, Groningen 9713 AV, The Netherlands; Department of Genetics, University of Groningen, Antonius Deusinglaan 1, Groningen, Groningen 9713 AV, The Netherlands; Department of Genetics, University Medical Center Groningen, Antonius Deusinglaan 1, Groningen, Groningen 9713 AV, The Netherlands

## Abstract

In this report, we analyse the use of virtual reality (VR) as a method to navigate and explore complex knowledge graphs. Over the past few decades, linked data technologies [Resource Description Framework (RDF) and Web Ontology Language (OWL)] have shown to be valuable to encode such graphs and many tools have emerged to interactively visualize RDF. However, as knowledge graphs get larger, most of these tools struggle with the limitations of 2D screens or 3D projections. Therefore, in this paper, we evaluate the use of VR to visually explore SPARQL Protocol and RDF Query Language (SPARQL) (construct) queries, including a series of tutorial videos that demonstrate the power of VR (see Graph2VR tutorial playlist: https://www.youtube.com/playlist?list=PLRQCsKSUyhNIdUzBNRTmE-_JmuiOEZbdH). We first review existing methods for Linked Data visualization and then report the creation of a prototype, Graph2VR. Finally, we report a first evaluation of the use of VR for exploring linked data graphs. Our results show that most participants enjoyed testing Graph2VR and found it to be a useful tool for graph exploration and data discovery. The usability study also provides valuable insights for potential future improvements to Linked Data visualization in VR.

## Introduction

In recent years Linked Data has increased in popularity for representing complex analysis results, i.e. ‘hairballs’ of scientific knowledge, in particular in light of the desire to increase the FAIRness of research results (2, 3).

In 2009, Sir Tim Berners Lee, who is also known as the ‘inventor of the World Wide Web’ because he invented the Hypertext Transfer Protocol protocol and Hypertext markup language for web pages, gave a famous Ted talk about Linked Data in which he described how incompatible data formats and documentation systems make it necessary to examine each data element in order to create something new (4, 5). Berners Lee suggested that uploading unadulterated raw data to the web as Linked Data would make it easier to combine, link and reuse existing data. Since then, shared vocabularies and ontologies have increasingly been used to structure data, and chemical and biological registries, as well as governments, have started using Linked Data to handle the large amounts of data they store (6–9).

The Resource Description Framework (RDF) developed by the World Wide Web Consortium is a standard for describing data on the web in a machine-readable format (10). The RDF data model describes information as subject–predicate–object relationships called triples. This kind of data can be queried using the SPARQL query language; however, the powers of SPARQL and linked data are not readily accessible to users unfamiliar with SPARQL. Graphical tools provide visual user interfaces to support the user in visually exploring and accessing this kind of data. These triples can be used to create a network graph visualization by representing the subject and object of each triple as nodes and the predicates as edges between them. Nodes that represent the same resource are merged in the visual representation.

Immersive technologies such as virtual reality (VR) and augmented reality (AR) are increasingly used in health and life sciences for a variety of applications, including therapeutics, training, simulation of real-world scenarios and data analysis for, e.g. genomics and medical imaging (11–15). Immersive analytics has established applications for abstract and multidimensional data in this domain (see (12) for a review). A limited number of applications exist to explore knowledge graphs in VR (16–18), but these are merely visualizations and do not offer many options for users to query and interact with the data.

We hypothesize that VR will allow users to more readily explore, compare and query large knowledge graphs using a gesture-driven interface that requires less technical expertise. In this VR context, users can use ontologies to search, order and filter data to their needs. Instead of writing SPARQL queries, the user can expand existing connections and define patterns in the data interactively using the VR controllers. Overall, VR adds a third dimension and an open space that can help users perform complex data analysis.

In this paper, to test this hypothesis, we first review recent visualization methods and tools used for the exploration and analysis of semantic web knowledge graphs and identify best practice methods for visualizing and interacting with SPARQL query results. We then select methods and materials and implement an experimental VR prototype to explore Linked Data and Graph2VR, evaluate its usability and investigate the human and VR environment factors that could enhance the exploration and analysis of semantic web knowledge graphs.

## Related work

As a basis for the Graph2VR experiment, we reviewed existing tools that provide best practice methods for addressing specific challenges when working with (large) graph databases. Our review included tools for visualizing graphs in 2D, 3D and VR; those working with ontologies, SPARQL and graph databases; backends for graph databases and general ways to visualize data in VR (Supplementary Appendix Table A1).

Later, we describe some notable tools, LODLive, GraphDB, Vasturiano, Toran, Visual Notation for OWL ontologies (VOWL), Gruff and Tarsier (18–25), that all provide rich graph exploration functionality, enumerate functional challenges and visualization aspects and highlight their implementation in Graph2VR. We also refer the reader to some overview papers about tools to visualize and interact with ontologies, Linked Data or graph databases in general, which have been tested and described, e.g. in the following papers. (6, 26, 27).

**Figure 1.**
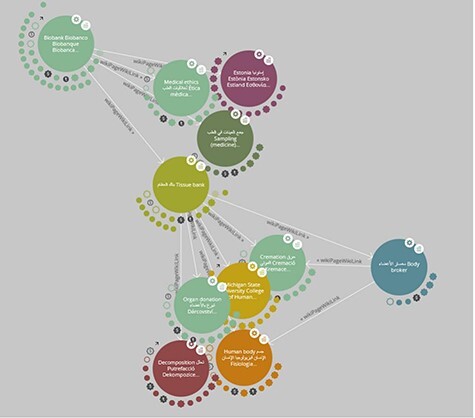
A screenshot of LodLive (19): the graph can be expanded by clicking on the small clouds or circles around the node.

In ‘LodLive’, the user is first asked for a SPARQL Endpoint and a Unique Resource Identifier (URI). Each node is represented as a circle in LodLive, and outgoing connections are represented as small nodes around it (see Figure 1). Each node offers some options in the form of a menu that includes ‘i’ for information, a button to focus on the node and to close other relationships, a button to open a link to the URI to show an online resource, a button to expand all relationships around that node and a button to remove the selected node. Once a node is added, existing connections to other open nodes are also displayed. However, beyond opening new connections and a comprehensive info panel that shows information about the respective node in a flexible way that makes use of different kinds of semantic annotations, integrating images and even google maps, the query possibilities in LodLive are quite limited.

**Figure 2.**
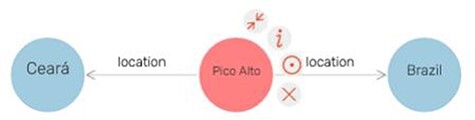
A small ‘Visual Graph’ in GraphDB (20). The node in the middle has a menu with the option to collapse connections around that node.

‘GraphDB’ and ‘Metaphactory’ are commercial software tools (20, 28). They use an internal database and offer an autocomplete search function to find URIs. The user is thus not required to know the precise URI, which makes the tools quite user-friendly. GraphDB also offers an option to collapse all nodes around a specific node instead of only allowing users to delete specific nodes (Figure 2).

**Figure 3.**
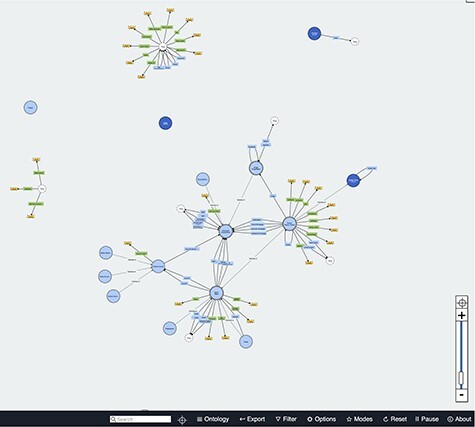
Screenshot of WebVOWL showing parts of the FOAF ontology (32).

The Visual Notation for OWL Ontologies ‘VOWL’ (22, 23) adds use of colour-blind-friendly colours and symbols to visualize different types of nodes and edges. Two examples of the implementation of VOWL are a plugin for Protégé and WebVOWL (29, 30). WebVOWL is an online visualization for ontologies. By default, WebVOWL starts with a subgraph of the Friend of a Friend Ontology (FOAF) ontology, as shown in Figure 3, but users can upload their own, owl files. QueryVOWL, another VOWL tool, can be used to query a SPARQL Endpoint using a textual search to create a visual graph as output (31).

Gruff is a commercial tool that can use its internal graph database (Allegro graph), SPARQL Endpoints and Neo4j. After selecting a database, users can ‘Display some sample triples’ from that database without any prior knowledge about the content, which is very convenient for new users. Gruff offers a visual query view to describe the necessary relations using variables and searching for nodes. These relations can contain specific URIs, such as constant nodes and variables. The relationships created in query view are then translated into an editable SPARQL query, which can be altered to add additional filters or other specific SPARQL commands. This translation from a visualization to an editable SPARQL query is convenient and makes Gruff stand out as a tool. However, screen size is a limiting factor for Gruff. When zooming out, the text becomes too small to read and is hidden. The user can move the window of visible nodes in all directions. Instead of using labels, Gruff uses colours and shapes to indicate similar nodes and repeating edges.

**Figure 4.**
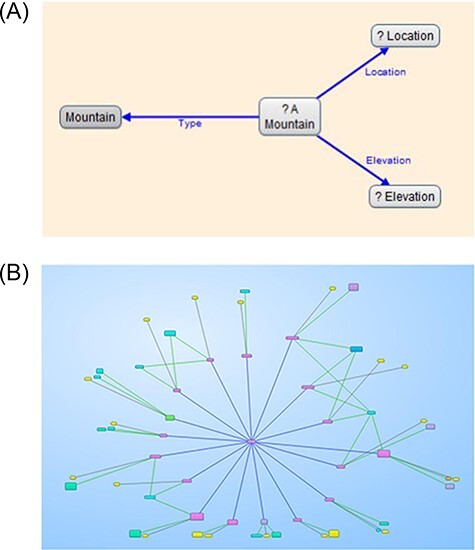
Gruff allows users to build queries visually and shows the SPARQL query, so it can be modified (4(A)). The results can be displayed as a visual graph (4(B))(24). (A) Screenshot of a query built-in Gruff’s ‘Graphical Query View’. (B) Screenshot of the resulting graph in Gruff’s ‘Graph View’. When zoomed out, the texts disappear, but the colours still indicate similar nodes and edges.

**Figure 5.**
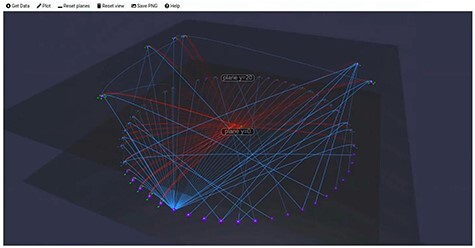
Tarsier filter data and can shift datapoints to different semantic planes accordingly. (This screenshot was taken from a video from the authors of Tarsier (33)).

Finally, ‘Tarsier’ is a tool that makes use of a 3D representation of SPARQL queries. Filters allow users to select nodes that fulfil specific criteria and shift them to another ‘semantic plane’. This tool was built with the intention to help new students to learn SPARQL and understand how filters work (Figure 5).

## Materials and Methods

Based on the analysis of existing tools and approaches, we prioritized a list of key features for Graph2VR, which are summarized in Table 1. We aimed to combine some of the strengths of the different methods from different tools in one VR application that enables immersive 3D visualization that allows interaction with and manipulation of data. Graph2VR is an extensive usable prototype that still has a few issues that need to be resolved. It is extendable and demonstrates how a VR application can be used to visualize and interact with Linked Data. A more detailed description of its features is present in the user manual in (34) and in five tutorial videos (1). Later, we describe the methodological considerations for these features in detail, grouped by initialization, visualization, navigation and data analysis.

**Table 1.** Key features of Graph2VR

Category	Description
Initialization	A configuration file is used to preconfigure which SPARQL Endpoint to start from and additional ones to switch to. Graph exploration usually starts from either a single URI or a small graph, which can also be specified in the configuration file. Image predicates, predicates that get suggested for new connections and colour coding can be adjusted in this file. We adopted the VOWL colour schema as a reasonable default.
Visualization	To visualize Linked Data, nodes are represented as spheres and the edges between them as arrows. The colour schema is used to represent different kinds of nodes or their current status (e.g. being selected or part of a query). A choice of graph layout (3D, 2D, hierarchical and class hierarchy) for the visualization will give the most flexibility for the user. Labels and images for nodes and labels for edges are preloaded when the graph is expanded.
Interaction	Graph2VR provides features for interacting with the visualized Linked Data. The user can grab and move single nodes or the whole graph. Once grabbed, the whole graph can be moved, rotated and scaled. There are four different graph layout options: 3D, 2D, hierarchical and class hierarchy. The user has different ways to navigate in the VR environment. Graph2VR is a room-scale VR application, so looking around and taking a few steps can help to move small distances. For larger distances, the user can either teleport or fly. Pinning nodes prevents them from being affected by the layout algorithm so that they can be restructured manually. The user can create new nodes, edges and graphs; drag and drop nodes and graphs; and convert nodes or edges to variables to create queries. New nodes can be added by searching for them in the database or by spawning a new variable node. After selecting single nodes or edges, the circle menu shows options for the selected node or edge for further interaction.
Data analysis	Graph2VR provides features for querying SPARQL Endpoints, including graph expansion. In addition, the nodes and edges can be used to generate custom queries visually. Triples can be selected to be part of a SPARQL query. A predefined set of commands, including selecting triples, creating variables, OrderBy and Limit, can be used to create a SPARQL query. Options that affect a whole triple can be found in the edge menu.

### Initialization

In many visualization tools, graph exploration starts from either a small subgraph (bottom-up or local view) or a global hierarchy (top-down or global view) in which zoom and filters are used to request more details (35). Starting from a subgraph, either an overview graph or a start node, the user can expand the graph by opening further connections. Commonly, tools that work with SPARQL need the Uniform Resource Locator of a SPARQL Endpoint to determine to which graph database their requests should be sent. Additionally, they need a URI, SPARQL query or keyword to know where to start the graph exploration. The graph can then be expanded further to explore it incrementally. In Graph2VR, one can start with an initially provided SPARQL query and explore from there.

### Visualization

This section summarizes methods around visualization, in particular use of layout, colour and information display.

#### Layout

In general, the layout of graphs in the tools is either static or is restructured over time to increase the distance between the nodes and the readability. The Fruchterman–Reingold algorithm is a force-directed graph algorithm (36). The regular runtime of the algorithm has a runtime of $\mathcal{O}(|N|^2 + |E|)$, where *N* is the number of nodes and *E* is the number of edges (37). The algorithm also works in three dimensions. As the graphs get larger, the number of node–node interactions increases. For larger graphs, other algorithms like the Barnes–Hut algorithm scale better. The Barnes–Hut algorithm combines the gravity centre of nodes that are further away so that fewer calculations need to be executed (38, 39). There are, of course, many more algorithms like Kamada–Kawai that could be used to handle even more nodes in a graph at once (36, 40). In a 3D representation, we can use the third dimension to compare a stack of 2D layers. In the literature, this kind of representation is known as semantic planes (25, 41), and Tarsier already makes use of it. Tarsier runs on a local server and can be accessed via its web interface. It allows the user to apply filters, then takes all the nodes that fulfil the selection criteria and shifts them to another semantic plane. A stack of different planes of information would allow a user to compare them without losing their internal structure (e.g. a tree structure). In this way, the user can, e.g. compare data and annotate the similarity between two resources. For comparisons, different predicates can be used to describe the kinds of connections between different nodes. In their Scientific Lens paper, Batchelor et al. name four predicates with decreasing similarity levels to compare similar entities that could be used to compare different entities with similar meanings: ‘owl: sameAs’, ‘skos: exactMatch’, ‘skos: closeMatch’ and ‘rdfs: seeAlso’ (42). In the final version of Graph2VR, we added those as predefined predicates so that a user can quickly adjust the predicate of an edge. In Graph2VR, results can be shown as a series of semantic planes representing the different results that match a given query pattern (see section Demonstration Data). These planes are useful for comparing different 2D structures and creating new connections. For example, multiple 2D layers of class hierarchies allow users to compare between different ontologies, which can be used to find similarities and differences.

#### Colour

The colouring of nodes and edges also varies from tool to tool. LodLive uses random colours, whereas Gruff reuses the same colour for the same types of nodes and edges. The VOWL colour scheme defines the colour of each node based on its properties, using specific colours for classes, variables, blank nodes and literals. It has also been designed to be colour-blind-friendly and understandable when printed in black and white (32). VOWL recommends using specific shapes or patterns to indicate different attributes, e.g. a ring around a node to represent a class. For the 3D environment, the colours can be reused, but some forms need to be adjusted. In 3D, for example, a circle around a sphere to indicate a class could be represented as either a circle or a sphere. Using different forms and shapes would also be a viable option to represent an object in Linked Data. In Graph2VR, we primarily apply the VOWL schema but have not yet implemented all of it. We also add some colours for variables and selected triples. Further details can be found in the manual (34). As graphs become larger through expansion, it becomes more important to be able to quickly identify different types of nodes, i.e. is the node a URI, a literal, a blank node or a variable? Some tools do not distinguish, displaying all nodes in the same or a custom colour. One example of such a tool is ‘Noda’, an application sold on Steam that allows the user to interact with 3D mindmaps or graph structures by adding nodes and edges with different sizes, symbols, colours and labels (43). Gruff colours the same kinds of connections in the same randomly chosen colour, whereas the VOWL colour scheme can help users make distinctions quickly (23). In Graph2VR, parts of the VOWL schema are applied, making literals (strings and numbers) yellow, URIs blue and classes light blue (only if the class relationship is expanded). Blank nodes are dark. When a node or edge is selected using the laser, it turns red. When converted to a variable, nodes and edges turn green. Nodes and edges glow white when the user hovers over them with the laser. If triples are part of the selection for a query, they shine in a bright yellow colour. While common edges are black with a black arrowhead, the arrowhead changes the colour for the ‘rdfs: subClassOf’ relationship to a white arrowhead.

#### Information display

One useful feature in tools like LODLive or WebVOWL is an information box containing essential information about a selected node, although which information is displayed varies. Typical information provided is the URI, label, class relationship and sometimes comments. SPARQL offers the ‘describe’ function to request some basic information, but the results can be quite extensive. Therefore, it is better to restrict the information to a selection of predicates.

To visualize query results, we parsed the results and displayed the URIs or, if findable, the labels of the nodes and edges in the graph. To further improve readability, the labels above the nodes are continuously rotated towards the user, as are the images. This can be seen in the first part of the Video tutorial (1). In contrast, the texts above the edges always sit on top of the connecting lines and are readable from both sides of the edges. Since URIs are often long strings of characters, the labels of the URIs are displayed instead (if available). The complete URI appears when the user hovers over the edge with the laser.

### Interaction

This section summarizes features for interaction with the visualization, in particular methods for controls, navigation, zooming and rotation and menus in VR.

#### Controls

To enable users to reorder nodes, we implemented a gesture-driven interface that allows the user to drag and drop them using the controllers. The user grabs a node with both hands while pressing the grip button. Grabbing a node while pressing the trigger button allows new links between two nodes to be created.

When multiple graphs exist in VR, it can be challenging to grab and manipulate the desired graph. To differentiate between different graphs, we implemented a sphere around all the nodes of a graph that is only visible from the outside of the graph, to avoid cluttering the view. This sphere determines the size of a graph and allows the user to interact with a specific graph. When a SPARQL query is executed and multiple results are created in different layers (semantic planes), each layer is generated as a separate graph. Grabbing and moving a graph enable the user to work with a specific subgraph. To remove less interesting graphs, we made it possible to delete sibling graphs (all the other graphs created by the same query aside from the current one) or all child graphs of a particular graph. When the semantic planes are displayed next to each other, we first tested drawing flat planes around the 2D visualizations. Those planes were transparent, similar to the sphere surrounding the 3D version of the graph. However, as multiple partially transparent 2D layers behind each other turned out to be more distracting than useful, we turned off the planes by default.

#### Navigation

We added a platform to Graph2VR to make orientation easier as it feels more natural for a user to stand and walk on a surface than to float in empty space. Common ways to move in room-scaled VR applications are moving in the room, walking with a thumbstick or trackpad, teleportation and, in some cases, flying. In Noda, users can drag themselves through the room. In GraphXR, users can rotate the whole graph. Finally, in 3D Force Graphs, the user can fly through a universe of huge nodes (17, 18, 43). To give the user maximum freedom for navigation, we implemented both flying and teleportation and, if the user looks around or moves, this also happens in the virtual environment.

#### Zoom and rotation

**Figure 6.**
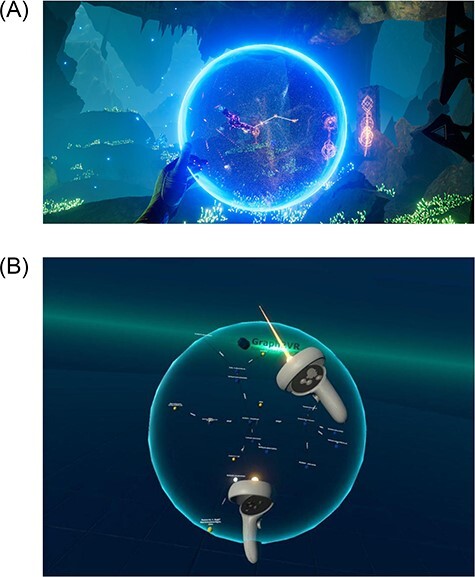
The sphere in Toran (6(A)) was the inspiration for having a sphere and for how to rotate, zoom and drag&drop a graph (21). For comparison, see the sphere in Graph2VR (6(B)). (A) Screenshot of Toran, a transparent sphere surrounds the game elements. (B) Screenshot of rotating a graph in Graph2VR. A transparent sphere surrounds the graph.

Graph2VR offers several ways to interact with a graph. When using the grip buttons of the VR controller on a node, the user can drag and drop a node close to the controller. Here, we were inspired by functions in the game/tech demo Toran (Figure 6(A)) (21). In that demo, a graph is embedded in a round sphere that can be freely rotated and zoomed (Figure 6(B)). The user can also create new connections between nodes within the sphere. As this also suits working with graphs, we implemented similar ways to interact with the graphs in Graph2VR. Both controllers’ grip buttons must be pressed close to the graph simultaneously to grab the whole graph, which can then be dragged around and rotated freely. When the controllers are moved together/away from each other, the graph is scaled down/up. It is possible to grab a node and the whole graph simultaneously. This results in scaling the entire graph; only the grabbed node does not scale with the graph, which can be used to scale specific nodes up or down. It is also possible to work with multiple graphs in Graph2VR, and each can have a different size.

### Circle menu

**Figure 7.**
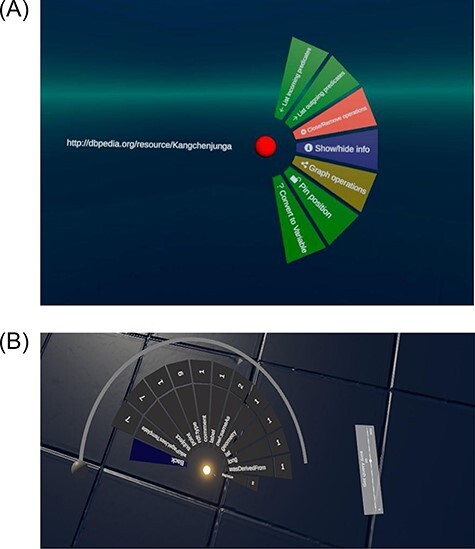
The circle menu in 7(A) shows different options based on the context. There are submenus that show more details, such as the outgoing connections in 7(B). (A) The circle menu after clicking on a node. (B) Circle menu displays multiple outgoing connections.

VR has not yet evolved to have standard control archetypes comparable to those used in 2D user interfaces. Nonetheless, users do need a system that allows them to quickly choose from sets of options. We were inspired to create a circular menu by the ‘Aesthethic Hover UI’ asset (44). Here, we first attempted to reuse the HoverUIKit, but it used depreciated packages, so we ultimately built our own circle menu. Most 2D tools like LOD Live, Gruff, GraphDB and Metaphactory show their menu and search options next to the nodes (19, 20, 24, 45), but VR offers a wider range of options. We therefore considered having the menu directly next to the node, on the left arm, on a virtual display or panel or static in front of the head as in a helmet display. After some testing, we determined that a menu with many options within a hairball of nodes would not be the appropriate solution and that the menu was best readable on the left controller. This circular menu has several submenus and shows the options that can be applied in the current context. When a node is selected, the menu displays options that can be applied to that node (Figure 7(A)). When selecting an edge, a menu for the edge is shown. In the node menu are further submenus that show all incoming or outgoing relations from or to the selected node. To improve the usability of the menu, we added some icons to the options in the circle menu. Click sounds indicate that buttons have been pressed, and white circles around newly spawned nodes that disappear within 2 s denote changes within the application. For new Graph2VR users, we have added a short help menu describing the main functionalities.

### Data analysis

This section summarizes methods for graph manipulation, in particular query generation, search, and save and load.

#### Query generation

In SPARQL, Select queries result in tabular answers, while Construct queries describe graph structures. Therefore, Graph2VR uses Select queries to populate the menu, e.g. with the incoming and outgoing connections of a node, and Construct queries to create graph structures. Nodes with the same URI are combined into one node in the visualization to generate a network graph instead of a list of triples. The submenu for incoming (and outgoing) connections lists the predicates pointing to (or from) the current node. They are then grouped by their predicates, and the number of available triples for each predicate is displayed next to the entry. When there are more predicates than can be displayed at once, a circular scrollbar can be used to scroll through the menu (Figure 7(B)). If a node with the same URI is added to a graph, it will be merged with the existing node. To restrict the number of results when expanding the graph (e.g. if one node has thousands of connections of the same type), there is a limit on how many connections to open (default is 25). This default limit can be adjusted using the limit slider below the menu (Figure 7(B)). The slider is not a linear scale but has fixed amounts of nodes.

To improve predicate readability, predicate labels are shown instead of the URI. If no label is available, the URI is shortened, but it is still possible to see the entire URI when hovering over the menu with the pointer. The predicates are ordered alphabetically by URI, not by the shortened version shown, which may confuse users when the base URI changes and the alphabetic order starts from the beginning again. When there are multiple connections between the same nodes, or in case of a self-reference, the straight edges are replaced by bent arrows to avoid overlapping texts.

#### Node removal

Once nodes are established, the user should have the option to remove them from the visualization. We implemented two different ways to do so. The simplest is the ‘close’ option, which removes the node and all triples containing this node from the visualization (but not the database). A more complex way to reduce the size of the graph is the ‘collapse’ option. This removes all leaf nodes around the selected node, while the node itself remains. Leaf nodes are nodes with no other connections within the visualization (but not necessarily in the underlying graph database). This helps reduce the number of nodes while preserving the graph structure.

For each node, the user has the option to remove it or to remove the surrounding leaf nodes (incoming, outgoing or both). When a node is deleted, the edges around it and its leaf nodes are also removed because there would be no triple left that contains them. This could lead to many single nodes that the user would have to remove manually. To prevent accidental removal when collapsing a selected node (when no triples are left containing it), and to keep newly created nodes that are not yet part of a triple, we decided to keep single nodes so that a user can connect them or start a new exploration from there.

When queries are sent in Graph2VR, the response will be displayed in new graphs that we call child graphs. To remove these again, each graph has the option to remove a whole graph at once. When a query creates multiple child graphs, it would be too much effort to remove those one-by-one. We therefore included an option to delete all child graphs from the original graph or the operation to delete all sibling graphs. Removing all child graphs will remove all child graphs of the respective graph. Each of the graphs also has the option to remove its sibling graphs, which will remove all unmodified sibling graphs created by the same query, leaving only the selected graph and modified graphs. If the last graph has been removed, it is possible to create a new graph by creating a new node, loading some saved data or using the search function(s).

#### Search

Graph2VR was intended to be more interactive than just a visualization. To be able to create some SPARQL queries visually, we added ways to create and rename variables or search for keywords. This required a text input system. We therefore reused a virtual keyboard from Unity’s asset store, VRKeys, that uses VR controllers as drumsticks to enter text (46). This approach to entering text in VR has two advantages over other applications, which mainly use a laser with a point-and-click system. Drumming the keyboard does not use an additional key, so it does not interfere with our controls, and it can be done with two hands simultaneously. We also implemented both a global search in the settings menu and a context-specific search that can be accessed from the node menu for variable nodes. In addition to the keyword, this second search function takes all the selected triples into account. Only results that match the selected variable in the given context and the keyword are shown. Both search functions attempt to perform an autocomplete search on the search term. Depending on the settings, the search can either be triggered with every keystroke on the VR keyboard or by pressing the return key.

#### Save and load

Graph2VR has two different ways to save and load data to a file. The first is to save the whole state of the application, including all the nodes, edges, their positions, labels, graphs, etc. Even images that have been loaded are saved, so they can be reloaded even if they are no longer on the internet. To prevent saved files from becoming too large, the image resolution is scaled down if it is too large. The quicksave option offers one save slot that will be overwritten every time. Alternatively, the user can specify a filename for a save state, allowing multiple save states. Loading a save state will overwrite the current session. Another way of saving and loading is to save triples as ntriples. This is a standard format that can also be read from other applications. In contrast to the first save option, this only saves the triples, and positions, images and single nodes are not stored. When loading an ntriples file, all the triples are automatically added to the current scene. In contrast to loading a save state, loading an ntriples file does not overwrite the current scene. Instead, all the triples are added as an additional graph. We strongly advise users not to load large ntriples files because Graph2VR could become slow or unresponsive. We could load about 5000 triples at once during our tests, but the framerate dropped to around five frames per second. For larger ntriples files, we recommend loading them into a SPARQL endpoint, e.g. a Virtuoso server, and accessing only the relevant parts from there (47).

## Implementation

Based on the methodological basis, we implemented a Graph2VR prototype in Unity (version 2 021.2f1) (48). This section summarizes implementation details, in particular use of Quest 2 VR, how to best represent RDF in unity, implementation of the layout algorithms and performance optimizations.

### Standalone on Quest 2 VR headset

Graph2VR was designed to run as a standalone version on the Quest 2 VR headset, but it also supports the HTC Vive headset. We recommend using the Quest 2 headset because its higher resolution provides better readability. Graph2VR can be compiled as a Windows application (.exe) or as a standalone application (.apk) for the Meta Quest 2 VR headset. Our Graph2VR implementation process started with the Free Unity WebXR Exporter from the Unity Asset store (49). This template includes a desert background and some objects and models for the VR controllers. To be able to select nodes, a laser pointer was added to the right controller. We used a sample dataset in a local Virtuoso server via docker as a database (50). To access the server, a modified DotNetRDF (version 2.6) is used (51, 52). At some point, DotNetRDF checks whether a specific interface is present, but even if it was present, the test failed in the Quest 2 standalone application. We resolved this by removing this check and recompiling the DotNetRDF library. One of the tougher decisions during the implementation process was whether to use SteamVR or OpenXR (53, 54). SteamVR, as commercial software, supports many VR headsets out of the box, provides 3D controller models and is, in general, easier to use due to its more abstract controller bindings. On the other hand, OpenXR would allow us to build a standalone application for the Quest2 headset that does not require a connection to a personal computer with a strong graphics card. Ultimately, we chose OpenXR and were able to create a standalone application for the Quest2.

### RDF representation

One issue we had to resolve was whether to use the representation of graphs provided by DotNetRDF, using iNodes and iGraphs, or whether to use the Unity in-memory representation for the nodes and edges. Unity-based prefabs can help to circumvent potential inconsistencies of two representations of the same dataset. Keeping both representations would be more error-prone but would allow reuse of more iGraph functionalities. The DotNetRDF representation has certain advantages when parsing and reusing the query results. Many functionalities were already present and utilizable. The disadvantage of this representation was that both the internal and the visual would need updates when adapting the graph. Additionally, while iGraphs in DotNetRDF support triples, adding a single node to our graphs, e.g. when a user adds a new node, led to differences between iGraph and visual representation. Consequently, the decision was made to rely on the unity-based representation to allow single nodes and enable step-by-step generation of new triples without consistency issues.

### Layout algorithms

**Figure 8.**
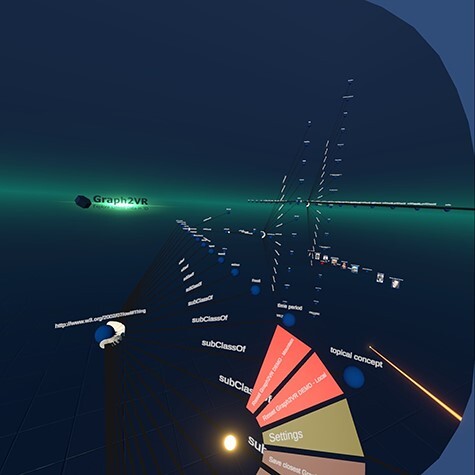
The hierarchical layout adds new triples, alternating vertical and horizontal on the ‘right’ side. Only the rdfs: SubClassOf relations are pointing to the ‘left’ side. They have a white arrowhead as specified in the VOWL schema.

Layout algorithms help to reorder the graph to improve readability. We implemented four layout algorithms: 3D force-directed, 2D force-directed, hierarchical view and class hierarchy. For the force-directed 3D layout, we used the Fruchterman Reingold Algorithm (37), which uses repulsive forces between nodes and attractive forces along the edges. Over time, the ‘temperature’ cools down, the forces get weaker, the adjustments in the graph get smaller and smaller and there can be a cut-off value. In Graph2VR, layout algorithms can be switched via the Graph operations menu. Inspired by Gephi, we modularized the layout algorithm, so the user can swap to other layout algorithms (55). For the 2D layout, we used a simple layout heuristic, simply ordering the nodes sequentially in a plane aided by minimal force direction. While the force-directed Fruchterman Reingold algorithm forms circles in 2D or spheres of nodes in 3D, the ‘Hierarchical View’ layout orders the nodes alternating in horizontal and vertical stacks (Figure 8). This makes it easier to read the labels of the nodes and find a specific node. Additionally, the nodes are sorted alphabetically in this layout. New outgoing nodes are usually added to the expanded node’s right side. However, an exception is the rdfs: subClassOf relationship, which points to the left.

**Figure 9.**
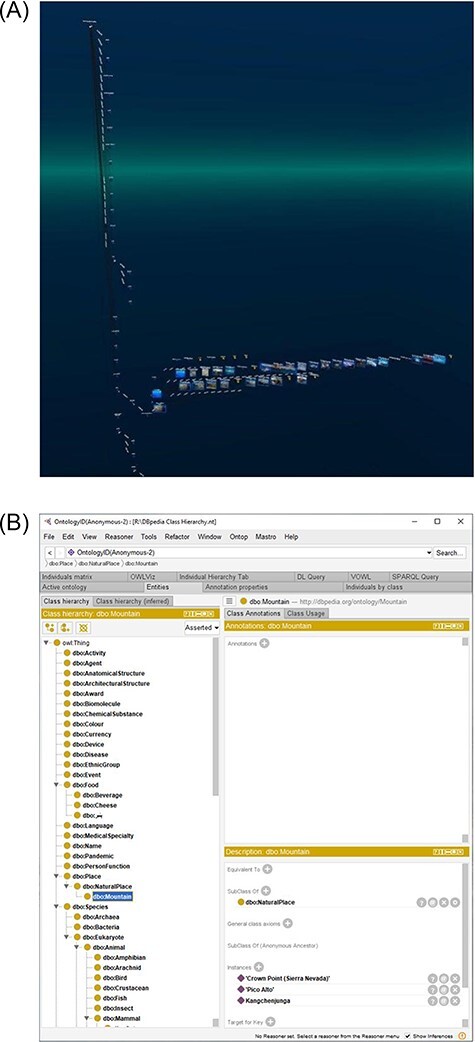
Comparison of the 3D class hierarchy in Graph2VR (9(A)) with the 2D class hierarchy in Protégé (9(B)) showing the same class hierarchy. (A) The class hierarchy in Graph2VR can display subclasses and individuals in a single 3D tree structure. The individuals and their attributes point in the third dimension. (B) The class hierarchy in Protégé is a tree structure starting at owl: thing. The individuals are displayed in a separate panel after selecting one of the classes.

Besides the ‘Hierarchical View’, we also added a ‘Class Hierarchy’ layout. The basic idea of this layout was to create a class hierarchy, like the tree structure in Protégé (29), but in three dimensions. The base of this layout is a 2D class hierarchy based on the rdfs: subClassOf predicate. Each of the classes (or subclasses) can contain multiple individuals of that class. We can use the extra dimension to display the individuals in a list orthogonal to the 2D class hierarchy based on the rdf: type predicate. See Figure 9(A).

Finally, a pin function was added to pin certain nodes to their current position. A pin prevents these nodes from being affected by the layout algorithm, but they can still be dragged around manually. This can have interesting effects when some nodes are pinned and layout algorithms are applied only to parts of the graph. It can be helpful to have a class hierarchy for the classes, pin it and continue exploring the individuals, e.g. with a force-directed algorithm.

### Performance optimizations

To prevent the whole application from stuttering while SPARQL queries are executed, we put them into separate threads. This way, Graph2VR will not stutter even if a query takes several seconds to be executed. The disadvantage of this is that the order of results from different queries is not determined. There is a race condition between the different queries, with the faster result displayed first. This may affect the search function when ‘search on key press’ is activated as it fires a query on each key press. This can be quite fast compared to the variation in execution times of the search queries, potentially leading to a situation where search results for an older but slower query overwrite newer results. To speed-up free-text search queries, we used the bif: contains command. This command is not supported by every SPARQL server but is supported by e.g. OpenLink’s Virtuoso (56). The bif: contains command triggers a search function on a preindexed internal Structured query language table. If such an index exists, this command can be used to speed up the free-text search. If the index does not exist, the server will most likely return empty results (57). During our tests, the DBpedia SPARQL endpoint supported the bif: contains command. One restriction of the bif: contains command (in Virtuoso) is that it only supports words with at least four letters and needs to be enclosed in brackets if it contains spaces. This is a problem if the search term consists of multiple connected words that belong together (e.g. names with ‘Mc’ at the beginning or ‘van’ in the middle). We did overcome this issue by separating the words but replacing the spaces of words with fewer than four letters with a star (any character) so that the whole name can be used as a single search term.

## Results

### Application overview

A SPARQL query can be used to define the initial graph. A combination of URIs, literals and variables in SPARQL is used to define query patterns and to request results that match this pattern from the database. Traditionally, formulating a SPARQL query involved manually searching for relevant URIs and predicates, a process that could take several minutes, especially for complex queries involving multiple triples. In Graph2VR, it is just a matter of expanding the graph by clicking on the desired predicate and expanding the graph and converting existing nodes (or edges) in the graph to variables. If necessary, new nodes and edges can be spawned. Once the relevant triples in the graph are selected, the results can be requested.

We implemented two different ways to display the query results. They can either be displayed as a single result graph containing all the triples merged into one new graph or as a series of separate result graphs stacked behind each other. These result graphs are independent graphs. To make sure that not all of them are moved, scaled and rotated at the same time, only one graph (the closest) can be grabbed at the same time. The relevant distance is the distance between the middle point of a graph and the left controller. There are also options to dispose of those stacks of graphs.

The idea of displaying parts of the results as stacked 2D projections had already been used in previous applications. Tarsier, for example, uses them to separate nodes that do or do not meet user-defined filter criteria into different planes (25). In the current version of Graph2VR, we did not implement a filter function, but the Tarsier approach might be an excellent way to do so in the future.

SPARQL has several commands to modify a query. The limit slider was already introduced to limit the number of nodes when expanding the graph (Figure 7(B)). It also can be used to limit the number of result graphs when sending a query. Another modifier is the ORDER BY command, which is used to order the stacked result graphs either descending or ascending. We added this menu option so that the variable can be selected by clicking on it, and there is a small adjacent button (ASC/DESC) to adjust the order.

Graph2VR can connect to local and online SPARQL Endpoints. If databases are preconfigured, users can switch between them via the circle menu.

### Demonstration data

For testing purposes, we provided an easy-to-understand data example from DBpedia, which is one of the best-known public sources for Linked Data and is based on Wikipedia (58).

In contrast to Wikipedia, it is possible to query data in DBpedia using SPARQL queries. One of the examples from a lecture about the semantic web was how to ask DBpedia for the second-highest mountain in a certain country, e.g. Australia (59). While it is challenging to find this information simply by reading Wikipedia articles, writing a SPARQL query to get this information from DBpedia is relatively easy. Since all the information is represented as triples, a user only needs to find out which predicates are used to encode height (dbo: elevation), location (dbp: location) and the fact that it should be a mountain (rdf: type dbo: mountain). The prefixes (res, dbo, dbp and rdf) are the abbreviations of the base URIs and are defined first. To find out which URIs need to be used, a user could open an entry about any mountain, look up the respective predicates and use that information to build their SPARQL query. The following example query is not about the second-highest mountain in Australia but rather about the highest mountains in DBpedia and their location. The mountains are ordered in descending order by height:

PREFIX res: <http://dbpedia.org/resource/>PREFIX dbo: <http://dbpedia.org/ontology/>PREFIX dbp: <http://dbpedia.org/property/>PREFIX rdf: <http://www.w3.org/1999/02/22-rdf-syntax-ns>
SELECT ?Mountain ?location ?heightWhere {?Mountain rdf: type dbo: Mountain.?Mountain dbp: location ?location.?Mountain dbo: elevation ?height.}ORDER BY DESC(?height)Limit 25

Within Graph2VR, this search can be done visually without manually looking up all the URIs for the predicates. All outgoing predicates are listed in the menu, so the user only needs to select them.

**Figure 10.**
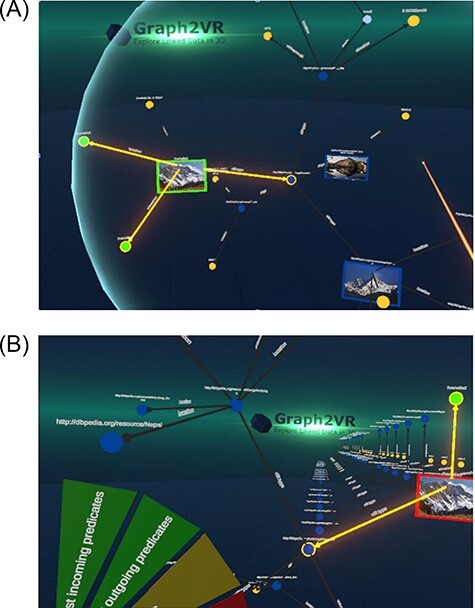
Creating a query pattern in Graph2VR (10(A)) and requesting the result graphs (10(B)). (A) Query patterns can be created visually in Graph2VR by selecting the triples in the graph that should be part of the query. The query can be modified using the Language settings, Order By options and the Query Limit slider. (B) After rotating the graph and clicking on ‘Request similar patterns’, the results are displayed in a stack of multiple independent graphs.

Within Graph2VR, it is possible to select the relevant triples, add those to the query pattern, transform the respective nodes into variables and then send the query to the SPARQL Endpoint. Besides the limit, the example query is the same one used as the example for Gruff (Figure 4). This task might still be tricky for a number of reasons: not every mountain’s height is represented using the same predicate, some mountains might be missing in the database or there might be multiple instances encoding the same mountain. However, this is a limitation of DBpedia data, not Graph2VR.

In many cases, DBpedia uses language tags that indicate the language used for the literal. Just asking for all labels results in many labels in different languages. We therefore added a language filter feature to Graph2VR that can be set via the settings menu. This allows users to request only labels with a specific language tag or no language tag at all. Once set, this is applied to every query. This may have consequences for the results, as it will not be indicated when the content is unavailable in the desired language or without a language tag. However, being able to set these conditions in the menu is more convenient than writing a SPARQL query to do so.

### Usability survey

**Figure 11.**
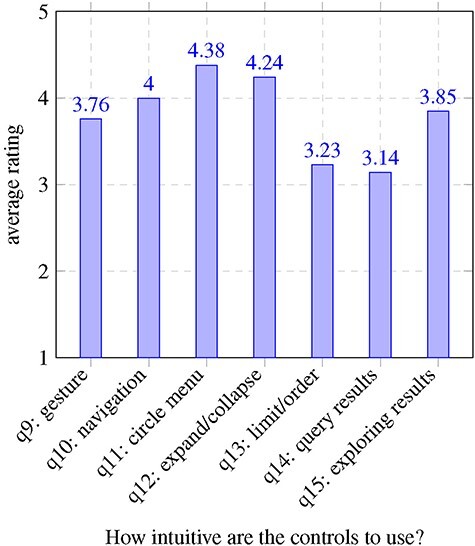
Results usability questionnaire questions 9–15.

**Figure 12.**
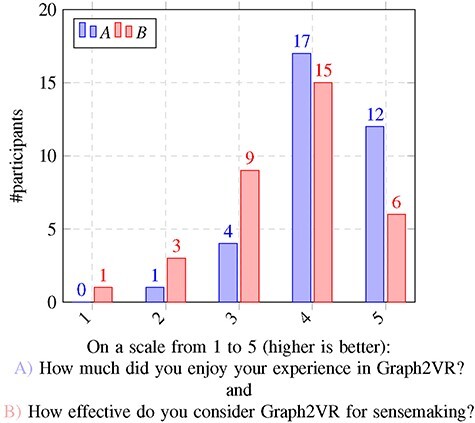
Results usability questionnaire questions 19 and 20.

To assess usability, we conducted a user evaluation with 34 participants. We were interested in how useful the application was for sensemaking, whether users enjoy the experience in VR and what influences their perceptions. By ‘sensemaking’, we mean the ability of the participant to answer questions or get understandable content from the semantic graph using the Graph2VR interface. We adopted this term from the Tarsier paper, which reused it from another earlier paper (25, 60) . Some participants already had some experience with in GraphDB, SPARQL or Linked Data, but others did not. We guided all first-time users through the application, told them about their options and let them test the different features. Then, we asked the users a series of questions, described in Supplementary Appendix B. The results are summarized in Figures 11 and 12 with data from the usability survey available in Supplementary Appendix B.

### Evaluation results

We identified several factors that influenced how much participants enjoyed testing Graph2VR and how effective they found the tool. One hypothesis was that people who are more used to playing computer games, especially if they have experience with VR glasses, might have an easier time using our application. It was uncertain whether age would have any effect, but we could imagine that younger participants, as digital natives, might find it easier. So there might be a weak correlation. To differentiate between enjoyment and effectiveness, both questions were asked after the VR experience and next to each other. We assumed that people who had a better experience, with fewer bugs, would rate their enjoyment as well as the tool’s effectivity for sensemaking more highly. We also expected a worse rating when the application had problems like poor readability or functions not working. An intuitive user interface makes the application more effective and enjoyable, whereas high complexity can make the application more interesting for experts but more difficult to use for beginners. During our studies, we determined that most participants found navigation and exploration quite easy, while the query-building seemed more challenging. Creating and modifying queries seemed to be noticeably easier for the ‘expert users’ who had at least some experience with Linked Data and writing SPARQL queries. Finally, we looked at the correlations between participants’ answers to the different questions. In addition to the Pearson correlation, *P*-values were calculated to determine the significance of those correlations. The complete correlation matrix is given in Supplementary Appendix Table A6. In Table 2, we summarize the statistically significant correlations.

**Table 2.** Interpretation of the statistically significant correlations of the usability study results ordered by p-values.

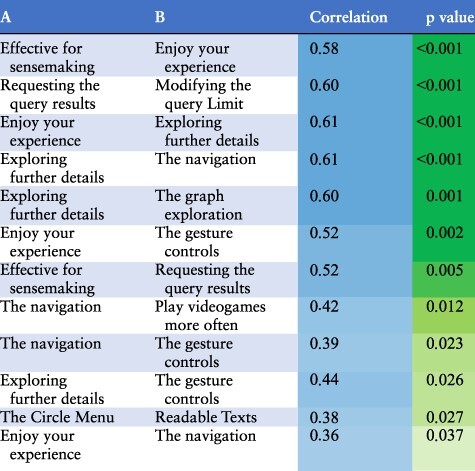

The questions, results and a more detailed evaluation can be found in Supplementary Appendix B. The findings are mostly as expected: having played video games more often is positively correlated with more intuitive navigation in the virtual world. Most participants also preferred flying to teleportation. We also found a positive correlation between navigation with gesture controls, requesting query results and exploring further details. We were curious whether age would have any significant effect on the experience of Graph2VR. Most participants (26/34) were between 30 and 50 years old. The sample size of people outside that age group was too small and probably not representative enough to draw any statistically significant conclusions. At the end of the experiment, we asked participants how much they enjoyed the experience and how effective Graph2VR is for sensemaking. Since both questions were asked at the same moment after the test, it is not a surprise that the answers are highly correlated. What is interesting is which factors are most relevant. For enjoying the experience, there is a strong correlation between the gesture controls and the graph exploration, as well as the exploration of further details. The exploration of further details is the second-to-last question during the VR session. A positive rating for this question implies that it was possible to request further results and that no major bug prevented this. When a problem occurred, the rating and the overall impression decreased. As Graph2VR was still under active development during the user study, some of the issues were fixed over time. Examples of this are the Limit slider that intersected with the scrollbar around the circle menu, nodes that were hard to select when they were zoomed too large and a layout option that led to many nodes being stapled at the same position.

Setting up the query, selecting the relevant triples, setting the order (and optionally the limit) and requesting the results seemed to be the most complex part of the study for many participants, especially those with no previous experience with SPARQL. Query-building was also the most complex and error-prone part as we were still developing features that interfered with this process. It also takes users time to really understand how this feature works, especially for individuals who did not have any experience with SPARQL queries. Setting up SPARQL query patterns (like in Gruff) is one of the most relevant features of Graph2VR. While it seemed to already be complex for inexperienced users, users who already had some experience were asking us to implement even more SPARQL commands.

## Discussion

Graph2VR is a prototype VR application for visualizing and exploring Linked Data in the form of 3D graphs. After exploring and testing multiple existing tools, we used Unity to create a user interface and DotNetRDF to connect to SPARQL Endpoints. We then tested our tool with a local Virtuoso server and DBpedia’s publicly accessible SPARQL Endpoint. To test the application and obtain feedback and suggestions for improvements, we conducted a usability study with 34 individuals who had never tried Graph2VR before.

### User feedback

Most testers were impressed and somewhat overwhelmed at the beginning, especially if they had never used a VR headset before. During the study, we guided them through the different functionalities and explained what they could do. During the tests, we noted their comments. After their VR session, we asked them to give feedback. This yielded valuable feedback from participants, including comments like ‘It felt good to be inside the world of the database’, ‘This is very fun to use and a great way of organizing and querying data’ and ‘I would love to try it out with other ontologies like Orphanet and connect it to applications like the RD-Connect Sample Catalogue (once that is in EMX2 so we can connect it)’ For explanation: The Entity Model eXtensible (EMX) is an internal metadata format of Molgenis, the current version is version 2 (61).

We explicitly asked the testers about specific problems they experienced and any suggestions they had for the application. It takes some time to explore all the different functionalities that we have built into Graph2VR. One tester mentioned, [I] “need to get used to the tool, but after that, it is good :)”. Another tester found the movement in space initially challenging: ‘moving in space was a bit hard in the beginning, but started to feel more natural along the way’. The most commonly mentioned issue among the testers was the readability of the text in the application. While the menu and nodes and edges nearby were legible, the labels of distant nodes and edges were hard to read. This problem is related to the resolution of the VR headset, which needs to display texts that are further away in just a couple of pixels. One general critique was that the VR headset is still quite heavy. After long test sessions, some people felt somewhat dizzy or tired. That was not unexpected. When using VR glasses, especially for the first time, manufacturers recommend taking breaks at least every 30 min. Our test sessions (including some introduction and filling in the questionnaire) took 40–60 min, on average.

We also asked more advanced users some additional questions, such as how Graph2VR compares to other conventional tools. Some of the experts mentioned that typing SPARQL queries by hand would still be faster than using the virtual keyboard and selecting the triples one-by-one. Graph2VR is somewhat limited by not having certain keywords, like Optional and Subqueries. Nevertheless, these users saw the potential that 3D visualization offers: ‘[Graph2VR is] much more fun, also allows much more data to be visualized, opens new possibilities’.

In all, we received constructive feedback, and we have already fixed several of the issues mentioned by testers. For example:

- The scrollbar no longer overlaps with the Limit slider.
- There was an issue with selecting nodes when the graph was scaled too big.
- Some testers asked for more visual feedback when clicking a button or to recognize when new nodes spawn. Both have been added.
- As we had at least two left-handed testers, we decided to add an option for left-handed people. It is not perfect as the menu still points to the right, but we have already received some positive feedback.

Other issues persist and need to be addressed in future versions:

- When the trigger is pressed while not pointing at any menu item, node,or edge, the menu should close. When scrolling down the scrollbar of the circle menu with the laser, it is easy to slip down the scrollbar, causing the menu to close. The circular scrollbar should be used instead via the slider knob on the scrollbar, a small ball that can be grabbed and then moved around.
- Some testers mentioned that the movement speed for flying in Graph2VR was quite fast and highly responsive, making it difficult to control the movement. This could be addressed in the future by adding an option to adjust the movement speed.
- When a graph bumps into the ‘floor’, the nodes collide with the floor and the graph deforms, which might result in a flat graph. This can also happen when a query is sent and a stack of result graphs is requested as the new graphs are spawned in the looking direction and might collide with the platform. One way to fix this is to trigger a layout algorithm.

Overall, user study feedback was valuable for identifying areas of improvement. Participants’ comments complemented their scores and elucidated specific strengths and weaknesses of Graph2VR. For example, several participants noted the difficulty of reading node and edge labels in VR, and this was also reflected in lower scores for readability. Participants with previous VR experience found navigation easier, while those who had experience with SPARQL queries found it easier to create the query patterns.

### Limitations of VR

One of our expectations was that the almost unlimited space in VR and 3D could help users visualize more nodes at once. Based on the reviewers’ comments, we can now confirm this. However, one ongoing challenge in VR is the readability due to a limited resolution of the node and edge labels. To improve readability, we increased the font size of texts when hovering on them, but they remain hard to read when the graph is too small or the text is too far away. We also replaced the URIs of nodes and edges with their labels. If no label is available, Graph2VR displays the Compact URI, a shortened version of the URI in which the namespace is replaced by a prefix to shorten the URI. In addition to the textual representation, images, colours and shapes can also help to differentiate between different types of nodes. We had originally planned to allow federated queries for requesting data from multiple SPARQL endpoints at once, but this has not yet been implemented.

### Ideas for future development

In the current version of Graph2VR, the colour schema is derived from the VOWL specification (30). But not all the colours and shapes from VOWL are implemented yet.

In the future, we would like to add further SPARQL commands to the application. Important SPARQL commands or keywords such as *OPTIONAL*, *SERVICE* (for federated queries), *UNION*, *BIND*, *OFFSET*, *DISTINCT*, *COUNT*, *GROUP BY* and *MINUS*, as well as *FILTER*, are supported by DotNetRDF but have not yet been implemented in the Graph2VR interface. In addition, an ‘undo’ button would be desirable, especially to restore accidentally deleted elements or graphs. Many test users found Graph2VR already quite complex, mainly because they were using it for the first time and did not have much experience with graph databases. Adding new functionalities will only increase the complexity further, so it might be a good idea to create a wizard to lead users through the query-building process in order to make this process easier. Another feature that should be implemented in the future is the ability to log and export some of the queries that have been performed. In addition, it should be possible to export the latest SPARQL query to use, e.g. in a browser. To find a specific object in the menu of outgoing nodes, another layer of entries would be helpful. This could be triggered when clicking the count of objects instead of the object itself. A similar feature would be to display all the existing connections between two nodes when setting the predicate for a new connection between two nodes. Finally, Graph2VR could be transformed into an augmented reality application in the future. This would especially be interesting as a multi-user application.

## Conclusion

We developed Graph2VR, a prototype VR application for visualizing and exploring Linked Data in the form of 3D graphs, and conducted a usability study with 34 testers, which provided valuable feedback on the tool’s usability and areas for improvement. We believe that Graph2VR represents a novel and engaging way of visualizing and exploring Linked Data. The overall user experience reported during the usability study was positive, especially among more experienced users. While there are still some limitations and issues to be addressed, we are confident that, with further development and refinement, VR will provide tools for working with large Linked Data graphs.

## Supplementary Material

baae008_Supp

## Data Availability

The source code of Graph2VR is openly available on GitHub http://github.com/molgenis/Graph2VR, under LGPL v3 license. The technical manual can be found in the attachments. The questions from the usability study, as well as the anonymous answers of the participants, can also be found in the attachments. A technical user manual for Graph2VR (version 1) can be found here: https://doi.org/10.5281/zenodo.8040594. A Graph2VR tutorial playlist is available on YouTube (1): https://www.youtube.com/playlist? list=PLRQCsKSUyhNIdUzBNRTmE-_JmuiOEZbdH.
