# Supplementary material for: Visualization and exploration of linked data using virtual reality
Source: Database (Oxford). 2024 Feb 23;2024:baae008. doi: 10.1093/database/baae008 (PMC11184448; doi:10.1093/database/baae008)
Supplement: baae008_Supp [file baae008_supp.zip › suppl_data/Appendix Tables.zip!/Table A.1_better_layout_version.docx]

**Program	Interesting for	Description	Special Feature	Year** last release **2D 3D VR	Linked Data (SPARQL) queries Ontology**

| Graph2VR | Visualisation, UI, Feature | Visualisation and interaction with Linked | Interactive VR GUI allowing exploration and | 2023 | 2023 | - | x | x | x | x | x |
| --- | --- | --- | --- | --- | --- | --- | --- | --- | --- | --- | --- |
|  |  | Data in VR | generating SPARQL queries visually in VR |  |  |  |  |  |  |  |  |
| Metaphactory [45] | UI | Plattform to explore and work with know- | Nice tool to interact with RDFS data | 2021 | 2023 | x | - | - | x | limited | x |
|  |  | ledge Graphs |  |  |  |  |  |  |  |  |  |
| VRNetzer [61] | Visualisation, UI | VRNetzer platform enables interactive net- | Extensive UI with many options, Flexible | 2021 | 2021 | - | - | x | - | - | - |
|  |  | work analysis in Virtual Reality | visualisation of huge networks in VR, many |  |  |  |  |  |  |  |  |
|  |  |  | ways to interact and analyse data |  |  |  |  |  |  |  |  |
| Noda [43] | Visualisation, UI | Allows creation of a 3D Mindmap | VR interaction with Data. Not necessarily | 2020 | 2020 | - | x | x | - | - | - |
|  |  |  | Linked Data, but with a nice UI |  |  |  |  |  |  |  |  |
| OntoTrek 3D [62] | Visualisation | A simple ontology terminology viewer | 3D tree structure of Ontology | 2019 | 2023 | - | x | - | x | - | x |
| Kineviz (GraphXR) [17, 63] | Visualisation, UI | Visualisation of data from Neo4j / CSV file | Visualisation of graph data in VR, SPARQL | 2015 | 2023 | - | x | x | NI | - | - |
|  |  | in VR | queries not possible |  |  |  |  |  |  |  |  |
| Tarsier [24] | Visualisation, UI, Feature | Visualisation of Semantic Planes | Semantic planes | 2018 | 2018 | x | x | - | x | x | x |
| ONVR / Cloud data visualisation ser- | Visualisation, UI | Visualisation of data in VR | Visualisation of data in VR | 2018 | 2021 | - | x | x | NI | - | - |
| vice ALAIRA [64] |  |  |  |  |  |  |  |  |  |  |  |
| Vasturiano [18] | Visualisation | Displays graph databases in VR | Displaying Graphs in VR | 2018 | 2023 | x | x | x | NI | - | - |
| Graph VR [65] | Visualisation | A tool to visualize and interact with three- | Visualisation of small network graphs in VR | 2018 | 2018 | - | x | x | - | - | - |
|  |  | dimensional graphs in VR. Not related to |  |  |  |  |  |  |  |  |  |
|  |  | Graph2VR |  |  |  |  |  |  |  |  |  |
| Big Data Visualization Platform for | Visualisation, UI, Feature | VR and AR Graph Visualization | VR and AR Compatibility, Voice control, | 2017 | NI | NI | x | x | NI | NI | NI |
| Mixed Reality [66] |  |  | state of the art, a lot of work went into op- |  |  |  |  |  |  |  |  |
|  |  |  | timisation |  |  |  |  |  |  |  |  |
| QueryVOWL [31] | Feature, UI | QueryVOWL is a visual query language for | Provides an autocomplete search in a web ap- | 2016 | 2015 | x | - | - | x | limited | x |
|  |  | Linked Data based upon the ontology visual- | plication for a graph database (DBpedia) |  |  |  |  |  |  |  |  |
|  |  | isation VOWL |  |  |  |  |  |  |  |  |  |
| GraphDB [20] | UI | Front- and backend to interact with graphs | Nice tool to interact with RDFS data | 2015 | 2023 | x | - | - | x | x | x |
|  |  | (rdf) |  |  |  |  |  |  |  |  |  |
| Gruff [23] | Feature | Graph Exploration and Editing | Many ways to interact with and visualize the | 2015 | 2023 | x | - | - | x | x | x |
|  |  |  | data, compatible with SPARQL and Neo4j |  |  |  |  |  |  |  |  |
| OSNAP [67] | Visualisation, UI | Graph visualisation software initially de- | VR, nice layouts | 2015 | 2014 | x | x | x | x | - | x |
|  |  | signed for flexible composition of 2D and 3D |  |  |  |  |  |  |  |  |  |
|  |  | ontology layouts |  |  |  |  |  |  |  |  |  |
| VOWL (Protégé plugin) [22, 31, 32] | Feature, Visualisation, UI | Protégé VOWL is a Protégé plugin for the | Colour and shape schema for colourblind, | 2014 | 2014 | x | - | - | x | x | x |
|  |  | user-oriented visualization of ontologies. | good choice which information to show when |  |  |  |  |  |  |  |  |
|  |  |  | selecting a node |  |  |  |  |  |  |  |  |
| WebVOWL [32] | Visualisation, UI | Web interface for Exploring and Editing | Web-based | 2014 | 2019 | x | - | - | x | limited | x |
| OpenGraphiti [68, 69] | Visualisation | A free, open source 3D data visualization en- | Visualisation of network traffic | 2014 | 2016 | x | x | experimental | x | NI | x |
|  |  | gine for data scientists to visualize and work |  |  |  |  |  |  |  |  |  |
|  |  | with semantic networks. |  |  |  |  |  |  |  |  |  |
| Haylyn [70] | Visualisation, Middleware, UI | Collaborative 3D Semantic Web Visualiza- | Early visualisation of Linked Data as graph | 2014 | NI | - | x | x | x | NI | x |
|  |  | tion and Analytics |  |  |  |  |  |  |  |  |  |
| LOD Live [19, 71] | UI | Frontend to visualize data from a SPARQL | Visualisation | 2012 | 2019 | x | - | - | x | - | - |
|  |  | endpoint |  |  |  |  |  |  |  |  |  |
| RelFinder [72] | Algorithm, UI | Frontend to find relationships within a | Nice Pathfinding and attractive UI, Asyn- | 2007 | 2012 | x | - |  | x | - | - |
|  |  | SPARQL Endpoint | chronous SPARQL query results lets means |  |  |  |  |  |  |  |  |
|  |  |  | user does not have to wait |  |  |  |  |  |  |  |  |
| Gephi [55] | Visualisation | Visualisation and exploration of graphs and | Can visualize many formats in various ways | 2008 | 2023 | x | x | - | x | x | x |
|  |  | networks |  |  |  |  |  |  |  |  |  |
| Arena3D [73–75] | Visualisation, Feature | With Arena3Dweb, users can integrate mul- | 3D Visualisation in multiple Layers | 2008 | 2023 | NI | x | - | - | - | - |
|  |  | tiple networks in a single view along with |  |  |  |  |  |  |  |  |  |
|  |  | their intra- and inter-layer connections |  |  |  |  |  |  |  |  |  |
| OntoSELF [76] | Algorithm | ONTOSELF: A 3D ONTOLOGY VISUAL- |  | 2007 | NI | x | x | - | x | - | x |
|  |  | IZATION TOOL |  |  |  |  |  |  |  |  |  |
| Skyrails / Interactorium [77] | Visualisation, UI | An interactive graph visualization system | Visuals where everything is moving as in a | 2007 | NI | - | x | - | - | - | - |
|  |  | that resembles a video game. | game |  |  |  |  |  |  |  |  |
| Semantic Analytics Visualization | Visualisation, UI, Feature | Semantic analytics through D visualization | Quite early | 2006 | NI | x | x | x | - | - | x |
| [16] |  | ”Semantic Analytics Visualization” |  |  |  |  |  |  |  |  |  |
| OntoSphere3D [78] | Visualisation | A visualization tool for ontologies that uses a | Protégé Plugin, 3D Visualisation | 2005 | 2008 | - | x | - | x | x | x |
|  |  | 3-dimensional space |  |  |  |  |  |  |  |  |  |

Table A.1: Tools for graph visualisation. For additional tools, refer to:[6, 25, 26]

If we could not test the software or find the information in the literature this is indicated with NI (No information).
