# Supplementary material for: Visualization and exploration of linked data using virtual reality
Source: Database (Oxford). 2024 Feb 23;2024:baae008. doi: 10.1093/database/baae008 (PMC11184448; doi:10.1093/database/baae008)
Supplement: baae008_Supp [file baae008_supp.zip › suppl_data/Appendix Tables.zip!/Table A.1_easier_editable_worse_layout_version.docx]

| **Program** | **Interesting for** | **Description** | **Special Feature** | **Year** | **last release** | **2D** | **3D** | **VR** | **Linked Data** | **(SPARQL) queries** | **Ontology** |
| --- | --- | --- | --- | --- | --- | --- | --- | --- | --- | --- | --- |
| Graph2VR | Visualisation, UI, Feature | Visualisation and interaction with Linked Data in VR | Interactive VR GUI allowing exploration and generating SPARQL queries visually in VR | 2023 | 2023 | **-** | x | x | x | x | x |
| Metaphactory [44] | UI | Plattform to explore and work with knowledge Graphs | Nice tool to interact with RDFS data | 2021 | 2023 | x | - | - | x | limited | x |
| VRNetzer [58] | Visualisation, UI | The VRNetzer platform enables interactive network analysis in Virtual Reality | Extensive UI with many options, Flexible visualisation of huge networks in VR, many ways to interact and analyse data | 2021 | 2021 | - | - | x | - | - | - |
| Noda [42] | Visualisation, UI | Allows to create a 3D Mindmap | VR interaction with Data - not necessarily Linked Data, but with a nice UI | 2020 | 2020 | - | x | x | - | - | - |
| OntoTrek 3D [59] | Visualisation | OntoTrek is a simple ontology terminology viewer | 3D tree structure of Ontology | 2019 | 2023 | - | x | - | x | - | x |
| Kineviz (GraphXR) [60, 16] | Visualisation, UI | Visualisation of data from Neo4j / CSV file in VR | Visualisation of graph data in VR, SPARQL queries not possible | 2015 | 2023 | - | x | x | NI | - | - |
| Tarsier [23] | Visualisatio, UI, Feature | Visualisation of Semantic Planes | Semantic planes | 2018 | 2018 | x | x | - | x | x | x |
| ONVR / Cloud data visualisation service ALAIRA [61] | Visualisation, UI | Visualisation of data in Virtual Reality | Visualisation of data in Virtual Reality | 2018 | 2021 | - | x | x | NI | - | - |
| Vasturiano [17] | Visualisation | Displays graph databases in Virtual Reality | Displaying Graphs in VR | 2018 | 2023 | x | x | x | NI | - | - |
| Graph VR [62] | Visualisation | A tool to visualize and interact with three-dimensional graphs in VR - Even so the name is similar, this tool is not related to Graph2VR | Visualisation of small network graphs in VR | 2018 | 2018 | - | x | x | - | - | - |
| Big Data Visualization Platform for Mixed Reality [30] | Visualisation, UI, Feature | VR and AR Graph Visualization | VR and AR Compatibility, Voice control, state of the art, a lot of work went into optimisation | 2017 | NI | NI | x | x | NI | NI | NI |
| QueryVOWL [30] | Feature, UI | QueryVOWL is a visual query language for Linked Data based upon the ontology visualization VOWL | Provides an autocomplete search in a web application for a graph database (DBpedia) | 2016 | 2015 | x | - | - | x | limited | x |
| GraphDB [19] | UI | Front- & backend to interact with graphs (rdf) | Nice tool to interact with RDFS data | 2015 | 2023 | x | - | - | x | x | x |
| Gruff[22] | Feature | Graph Exploration & Editing | Many ways to interact with and visualize the data, compatible with SPARQL as well as Neo4j | 2015 | 2023 | x | - | - | x | x | x |
| OSNAP [64] | Visualisation, UI | Graph visualisation software initially designed for the flexible composition of 2D and 3D ontology layouts | VR, nice layouts | 2015 | 2014 | x | x | x | x | - | x |
| VOWL (Protégé plugin) [21, 31, 30] | Feature, Visualisation, UI | ProtégéVOWL is a Protégé plugin for the user-oriented visualization of ontologies. | Colour & shape schema for colourblind, good choice which information to show when selecting a node | 2014 | 2014 | x | - | - | x | x | x |
| WebVOWL [31] | Visualisation, UI | Web interface for Exploring and Editing | Web-based | 2014 | 2019 | x | - | - | x | limited | x |
| OpenGraphiti [65, 66] | Visualisation | OpenGraphiti is a free and open source 3D data visualization engine for data scientists to visualize semantic networks and to work with them. | Visualisation of network traffic | 2014 | 2016 | x | x | experimental | x | NI | x |
| Haylyn [67] | Visualisation, Middleware, UI | Collaborative 3D Semantic Web Visualization and Analytics | Early visualisation of Linked Data as graph | 2014 | NI | - | x | x | x | NI | x |
| LOD Live [18,86] | UI | Frontend to visualize data from a SPARQL endpoint | Visualisation | 2012 | 2019 | x | - | - | x | - | - |
| RelFinder [69] | Algorithm, UI | Frontend to find relationships within a SPARQL Endpoint | Nice Pathfinding and great looking UI, Asynchronous SPARQL query results lets the user barely wait | 2007 | 2012 | x | - | - | x | - | - |
| Gephi [54] | Visualisation | Visualisation and exploration of graphs and networks | Can visualize many formats in various ways | 2008 | 2023 | x | x | - | x | x | x |
| Arena3D [70, 71, 72] | Visualisation, Feature | With Arena3Dweb, users can integrate multiple networks in a single view along with their intra- and inter-layer connections | 3D Visualisation in multiple Layers | 2008 | 2023 | NI | x | - | - | - | - |
| OntoSELF [73] | Algorithm | ONTOSELF: A 3D ONTOLOGY VISUALIZATION TOOL | - | 2007 | NI | x | x | - | x | - | x |
| Skyrails / Interactorium [74] | Visualisation, UI | Skyrails is an interactive graph visualization system that looks a lot like a video game. | Visuals like in a game everything is moving | 2007 | NI | - | x | - | - | - | - |
| Semantic Analytics Visualization [15] | Visualisation, UI, Feature | Semantic analytics through D visualization "Semantic Analytics Visualization" | quite early | 2006 | NI | x | x | x | - | - | x |
| OntoSphere [75] | Visualisation | OntoSpere3D is a visualization tool for ontologies that uses a 3-dimensional space | Protégé Plugin, 3D Visualisation | 2005 | 2008 | - | x | - | x | x | x |

Table A.1: Tools for graph visualisation. For more tools see:[6, 25, 26]

If we could no test the software or or find the information in the literature this is indicated with NI (No information).
