# Supplementary material for: Visualization and exploration of linked data using virtual reality
Source: Database (Oxford). 2024 Feb 23;2024:baae008. doi: 10.1093/database/baae008 (PMC11184448; doi:10.1093/database/baae008)
Supplement: baae008_Supp [file baae008_supp.zip › suppl_data/Appendix Tables.zip!/Table B2.docx]

| 1 | I consent that my answers can be used for scientific research and the evaluation and improvement of Graph2VR. |
| --- | --- |
| 2 | Do you have a visual impairment other than short/long  sightedness e.g., limited field of view or colour blindness? |
| 3 | How old are you? |
| 4 | How often do you play video games on a computer or con-  sole? |
| 5 | Have you ever used Virtual Reality glasses before within  the last 5 years? |
