# Supplementary material for: Visualization and exploration of linked data using virtual reality
Source: Database (Oxford). 2024 Feb 23;2024:baae008. doi: 10.1093/database/baae008 (PMC11184448; doi:10.1093/database/baae008)
Supplement: baae008_Supp [file baae008_supp.zip › suppl_data/Appendix Tables.zip!/Table B3.docx]

| 6 | Do you have a sense of really being in the virtual world? |
| --- | --- |
| 7 | On a scale from 1-5, how readable do you consider the  texts in the application? |
| 8 | How would you rate your current level of nausea? |
| 9 | How intuitive are the controls to use?  The gesture controls (grabbing, zooming, rotation) |
| 10 | How intuitive are the controls to use?  The navigation in the visualisation |
| 11 | How intuitive are the controls to use?  The circle menu |
| 12 | How intuitive are the controls to use?  The graph exploration (expanding/collapsing) |
| 13 | How intuitive are the controls to use?  Modifying the query (Limit / Order By) |
| 14 | How intuitive are the controls to use?  Requesting the query result |
| 15 | How intuitive are the controls to use?  Exploring further details from the results |
| 16 | Comments during the demo? (optional) |
| 17 | How reactive was the application? |
