# Supplementary material for: Visualization and exploration of linked data using virtual reality
Source: Database (Oxford). 2024 Feb 23;2024:baae008. doi: 10.1093/database/baae008 (PMC11184448; doi:10.1093/database/baae008)
Supplement: baae008_Supp [file baae008_supp.zip › suppl_data/Appendix Tables.zip!/Table B4.docx]

| 18 | How familiar have you been with graph databases and/or  the SPARQL query language before this experiment? |
| --- | --- |
| 19 | On a scale from 1 to 5 - how much did you enjoy your  experience in Graph2VR? |
| 20 | How effective do you consider Graph2VR for sensemaking? |
| 21 | How easy was it to examine specific visual details of the  environment? Did you have problems with specific features? |
| 22 | Do you have any other comments or feedback? |
