# Supplementary material for: Visualization and exploration of linked data using virtual reality
Source: Database (Oxford). 2024 Feb 23;2024:baae008. doi: 10.1093/database/baae008 (PMC11184448; doi:10.1093/database/baae008)
Supplement: baae008_Supp [file baae008_supp.zip › suppl_data/Appendix Tables.zip!/Table B5.docx]

| 26 | How does VR compare to conventional methods? |
| --- | --- |
| 27 | How easy is the visualisation to formulate simple  SPARQL queries? |
| 28 | What should be done to make Graph2VR more useful to  your daily work? |
