# Supplementary material for: Visualization and exploration of linked data using virtual reality
Source: Database (Oxford). 2024 Feb 23;2024:baae008. doi: 10.1093/database/baae008 (PMC11184448; doi:10.1093/database/baae008)
Supplement: baae008_Supp [file baae008_supp.zip › suppl_data/Appendix Tables.zip!/Table B6.docx]

| Table B.6: For the evaluation of the user study results. we calculated the Pearson correlation between the answers and their p-values using SPSS. The values of the rating functionalities (questions 9-15) are inverted  (5 is very intuitive. 1 is very difficult) So the correlations are easier understandable. | | | | | | | | | | | | | | | | | | |
| --- | --- | --- | --- | --- | --- | --- | --- | --- | --- | --- | --- | --- | --- | --- | --- | --- | --- | --- |
|  | | How old are you? | How often do you play videogames on a computer or console? | Have you ever used Virtual Reality glasses before within the last 5 years? | Do you have a sense of really being in the virtual world? | On a scale from 1-5 how readable do you consider the texts in the application? | How would you rate your current level of nausea? | How intuitive are the controls to use: The gesture controls? | How intuitive are the controls to use: The navigation in the application? | How intuitive are the controls to use: The circlemenu? | How intuitive are the controls to use: The graph exploration? | How intuitive are the controls to use: Modifying the query Limit? | How intuitive are the controls to use: Requesting the query results? | How intuitive are the controls to use: Exploring further details? | How reactive was the application? | On a scale from 1 to 5 how much did you enjoy your experience? | How effective do you consider Graph2VR for sensemaking? |  |
| How old are you? | Pearson Correlation | -- |  |  |  |  |  |  |  |  |  |  |  |  |  |  |  |  |
|  | N | 34 |  |  |  |  |  |  |  |  |  |  |  |  |  |  |  |  |
| How often do you play videogames on a computer or console? | Pearson Correlation | 0,060 | -- |  |  |  |  |  |  |  |  |  |  |  |  |  |  |  |
|  | Sig. (2-tailed) | 0,738 |  |  |  |  |  |  |  |  |  |  |  |  |  |  |  |  |
|  | N | 34 | 34 |  |  |  |  |  |  |  |  |  |  |  |  |  |  |  |
| Have you ever used Virtual Reality glasses before within the last 5 years? | Pearson Correlation | -0,146 | 0,185 | -- |  |  |  |  |  |  |  |  |  |  |  |  |  |  |
|  | Sig. (2-tailed) | 0,411 | 0,294 |  |  |  |  |  |  |  |  |  |  |  |  |  |  |  |
|  | N | 34 | 34 | 34 |  |  |  |  |  |  |  |  |  |  |  |  |  |  |
| Do you have a sense of really being in the virtual world? | Pearson Correlation | 0,142 | -0,184 | 0,058 | -- |  |  |  |  |  |  |  |  |  |  |  |  |  |
|  | Sig. (2-tailed) | 0,422 | 0,299 | 0,743 |  |  |  |  |  |  |  |  |  |  |  |  |  |  |
|  | N | 34 | 34 | 34 | 34 |  |  |  |  |  |  |  |  |  |  |  |  |  |
| On a scale from 1-5 how readable do you consider the texts in the application? | Pearson Correlation | 0,100 | 0,112 | 0,260 | 0,275 | -- |  |  |  |  |  |  |  |  |  |  |  |  |
|  | Sig. (2-tailed) | 0,578 | 0,533 | 0,143 | 0,121 |  |  |  |  |  |  |  |  |  |  |  |  |  |
|  | N | 33 | 33 | 33 | 33 | 33 |  |  |  |  |  |  |  |  |  |  |  |  |
| How would you rate your current level of nausea? | Pearson Correlation | -0,116 | -0,243 | -0,090 | 0,037 | -0,197 | -- |  |  |  |  |  |  |  |  |  |  |  |
|  | Sig. (2-tailed) | 0,514 | 0,166 | 0,612 | 0,836 | 0,271 |  |  |  |  |  |  |  |  |  |  |  |  |
|  | N | 34 | 34 | 34 | 34 | 33 | 34 |  |  |  |  |  |  |  |  |  |  |  |
| How intuitive are the controls to use: The gesture controls? | Pearson Correlation | -0,104 | 0,042 | 0,130 | 0,136 | -0,032 | -0,003 | -- |  |  |  |  |  |  |  |  |  |  |
|  | Sig. (2-tailed) | 0,566 | 0,815 | 0,469 | 0,451 | 0,861 | 0,989 |  |  |  |  |  |  |  |  |  |  |  |
|  | N | 33 | 33 | 33 | 33 | 32 | 33 | 33 |  |  |  |  |  |  |  |  |  |  |
| How intuitive are the controls to use: The navigation in the application? | Pearson Correlation | -0,159 | 0,424 | 0,000 | 0,264 | 0,122 | 0,190 | 0,394 | -- |  |  |  |  |  |  |  |  |  |
|  | Sig. (2-tailed) | 0,368 | 0,012 | 1,000 | 0,131 | 0,500 | 0,281 | 0,023 |  |  |  |  |  |  |  |  |  |  |
|  | N | 34 | 34 | 34 | 34 | 33 | 34 | 33 | 34 |  |  |  |  |  |  |  |  |  |
| How intuitive are the controls to use: The circlemenu? | Pearson Correlation | 0,058 | 0,172 | 0,045 | 0,202 | 0,384 | 0,133 | -0,257 | 0,238 | -- |  |  |  |  |  |  |  |  |
|  | Sig. (2-tailed) | 0,744 | 0,329 | 0,800 | 0,252 | 0,027 | 0,453 | 0,150 | 0,175 |  |  |  |  |  |  |  |  |  |
|  | N | 34 | 34 | 34 | 34 | 33 | 34 | 33 | 34 | 34 |  |  |  |  |  |  |  |  |
| How intuitive are the controls to use: The graph exploration? | Pearson Correlation | -0,037 | -0,201 | -0,234 | 0,208 | -0,092 | 0,171 | 0,182 | 0,263 | 0,206 | -- |  |  |  |  |  |  |  |
|  | Sig. (2-tailed) | 0,836 | 0,255 | 0,182 | 0,239 | 0,611 | 0,333 | 0,310 | 0,134 | 0,242 |  |  |  |  |  |  |  |  |
|  | N | 34 | 34 | 34 | 34 | 33 | 34 | 33 | 34 | 34 | 34 |  |  |  |  |  |  |  |
| How intuitive are the controls to use: Modifying the query Limit? | Pearson Correlation | -0,036 | -0,056 | 0,301 | 0,338 | 0,081 | -0,065 | 0,162 | 0,315 | 0,038 | 0,330 | -- |  |  |  |  |  |  |
|  | Sig. (2-tailed) | 0,849 | 0,769 | 0,106 | 0,067 | 0,676 | 0,732 | 0,392 | 0,090 | 0,841 | 0,075 |  |  |  |  |  |  |  |
|  | N | 30 | 30 | 30 | 30 | 29 | 30 | 30 | 30 | 30 | 30 | 30 |  |  |  |  |  |  |
| How intuitive are the controls to use: Requesting the query results? | Pearson Correlation | 0,018 | 0,006 | 0,333 | 0,128 | 0,137 | 0,218 | 0,166 | 0,489 | 0,182 | 0,301 | 0,596 | -- |  |  |  |  |  |
|  | Sig. (2-tailed) | 0,929 | 0,974 | 0,084 | 0,515 | 0,496 | 0,266 | 0,398 | 0,008 | 0,354 | 0,119 | 0,001 |  |  |  |  |  |  |
|  | N | 28 | 28 | 28 | 28 | 27 | 28 | 28 | 28 | 28 | 28 | 28 | 28 |  |  |  |  |  |
| How intuitive are the controls to use: Exploring further details? | Pearson Correlation | -0,011 | 0,057 | -0,041 | 0,364 | -0,010 | 0,042 | 0,435 | 0,609 | 0,264 | 0,596 | 0,248 | 0,307 | -- |  |  |  |  |
|  | Sig. (2-tailed) | 0,958 | 0,783 | 0,843 | 0,067 | 0,963 | 0,839 | 0,026 | 0,001 | 0,192 | 0,001 | 0,223 | 0,127 |  |  |  |  |  |
|  | N | 26 | 26 | 26 | 26 | 25 | 26 | 26 | 26 | 26 | 26 | 26 | 26 | 26 |  |  |  |  |
| How reactive was the application? | Pearson Correlation | -0,064 | -0,309 | 0,119 | -0,092 | 0,047 | 0,153 | 0,189 | -0,076 | -0,186 | -0,059 | 0,139 | 0,320 | -0,144 | -- |  |  |  |
|  | Sig. (2-tailed) | 0,718 | 0,075 | 0,504 | 0,606 | 0,797 | 0,386 | 0,293 | 0,669 | 0,293 | 0,741 | 0,463 | 0,097 | 0,484 |  |  |  |  |
|  | N | 34 | 34 | 34 | 34 | 33 | 34 | 33 | 34 | 34 | 34 | 30 | 28 | 26 | 34 |  |  |  |
| On a scale from 1 to 5 how much did you enjoy your experience? | Pearson Correlation | -0,053 | -0,034 | 0,164 | 0,336 | 0,109 | -0,167 | 0,516 | 0,360 | 0,084 | 0,206 | 0,292 | 0,343 | 0,613 | 0,124 | -- |  |  |
|  | Sig. (2-tailed) | 0,766 | 0,850 | 0,355 | 0,052 | 0,548 | 0,346 | 0,002 | 0,037 | 0,639 | 0,242 | 0,117 | 0,074 | 0,001 | 0,484 |  |  |  |
|  | N | 34 | 34 | 34 | 34 | 33 | 34 | 33 | 34 | 34 | 34 | 30 | 28 | 26 | 34 | 34 |  |  |
| How effective do you consider Graph2VR for sensemaking? | Pearson Correlation | 0,145 | 0,010 | 0,159 | 0,268 | 0,095 | -0,120 | 0,038 | 0,119 | 0,211 | 0,202 | 0,183 | 0,516 | 0,263 | 0,231 | 0,576 | -- |  |
|  | Sig. (2-tailed) | 0,413 | 0,956 | 0,370 | 0,125 | 0,600 | 0,500 | 0,834 | 0,503 | 0,230 | 0,251 | 0,332 | 0,005 | 0,194 | 0,189 | 0,000 |  |  |
|  | N | 34 | 34 | 34 | 34 | 33 | 34 | 33 | 34 | 34 | 34 | 30 | 28 | 26 | 34 | 34 | 34 |  |
